# The POVM Theorem in Bohmian Mechanics

**DOI:** 10.3390/e27040391

**Published:** 2025-04-07

**Authors:** Christian Beck, Dustin Lazarovici

**Affiliations:** Humanities and Arts Department, Technion—Israel Institute of Technology, Haifa 3200003, Israel

**Keywords:** Born rule, quantum equilibrium, Bohmian mechanics, POVMs, quantum measurement theory

## Abstract

The POVM theorem is a central result in Bohmian mechanics, grounding the measurement formalism of standard quantum mechanics in a statistical analysis based on the quantum equilibrium hypothesis (the Born rule for Bohmian particle positions). It states that the outcome statistics of an experiment are described by a positive operator-valued measure (POVM) acting on the Hilbert space of the measured system. In light of recent debates about the scope and status of this result, we provide a systematic presentation of the POVM theorem and its underlying assumptions with a focus on their conceptual foundations and physical justifications. We conclude with a brief discussion of the scope of the POVM theorem—especially the sense in which it does (and does not) place limits on what is “measurable” in Bohmian mechanics.

## 1. Introduction

The POVM theorem is a central result in Bohmian mechanics, grounding the measurement formalism of standard quantum mechanics in the Bohmian theory of particle motions and its statistical analysis. As a consequence of quantum equilibrium—the Born rule for Bohmian particle positions—the theorem states that the outcome statistics of an experiment are described by a positive operator-valued measure (POVM) acting on the Hilbert space of the measured system. A special case of POVMs are projection-valued measures (PVMs), which correspond to the self-adjoint “observable operators” of textbook quantum mechanics.

The result is based on the analysis of measurements first outlined in Part II of Bohm’s 1952 paper [1] and further elaborated by Dürr, Goldstein, and Zanghì (DGZ), who proved the general theorem [2].

The basic idea is simple. A quantum measurement is an interaction between a (usually microscopic) target system and some macroscopic apparatus, whose final configuration (“pointer position”) indicates the measurement outcome. As a result of the interaction, the Schrödinger evolution leads to an entangled superposition of the system + apparatus wave function, schematically,

(1) $$\varphi \left(x\right)\Phi_{0}\left(y\right)⟶\sum_{k}c_{k}\psi_{k}\left(t,x,y\right),$$

where the components $\psi_{k}$ are localized in disjoint regions of configuration space, corresponding to different pointer readings. The outcome distribution can then be predicted from the Born rule applied to the relevant pointer configurations. It will be given by a quadratic form of the initial wave function φ prepared for the target system, which corresponds to a POVM.

Similar arguments may be familiar from other discussions of the quantum measurement process, including in standard quantum mechanics [3,4], where they arguably provide the most transparent way of understanding the status of (generalized) observables and indicate that their measurement statistics can be reduced to the Born rule for (pointer) positions. In fact, the key insight already underlies the development of the measurement formalism in von Neumann’s *Mathematische Grundlagen der Quantenmechanik* (1932):

In a measurement, we cannot consider the system S in isolation; rather, in order to mathematically trace its interaction with a measuring device M, we must examine the system S+M. The theory of measurement is, after all, a statement concerning S+M, as it is meant to describe how the state of S is related to certain features of the state of M (namely, the positions of certain pointers, which the observer reads off).

([5] p. 187; our translation.)

As we will discuss in more detail, Bohmian mechanics turns these considerations into a robust analysis of measurements and their statistical description. The flip side is that the resulting theorem also places rigorous constraints on the kind of measurement statistics that Bohmian mechanics could predict, thereby frustrating certain attempts at deriving experimental predictions (specifically about arrival times, see [6,7] and the critique in [8,9]) directly from microscopic Bohmian trajectories while ignoring the active role of the measurement process.

This is the reason—or at least the trigger—for a controversy about the scope and status of the POVM theorem that has erupted in recent years (see, in particular, [10] contra [9]). While this controversy and skepticism about the POVM theorem is, in our view, unwarranted, it has also raised legitimate questions that are worth addressing in detail. These include:

1. What is the physical status of the mathematical assumptions underlying the POVM theorem and its applications?
2. Given that the Bohmian theory involves no laws or postulates about ”measurements”, how can it imply such a strong result about them?
3. Is the POVM theorem a result about what quantities are and are not *measurable*?
4. Does the theorem imply the *empirical equivalence* of Bohmian mechanics and standard quantum mechanics?

In this paper, our focus lies on the first question (on which all others depend). In the existing literature (e.g., [2,9,11,12]), the Bohmian POVM theorem is typically presented as a precise but rather abstract mathematical result, while its application to real-world experiments usually relies on additional, often implicit, assumptions. We seek to make those explicit by distinguishing between the basic theorem (Theorem 1) and a more concrete implementation (our Theorem 2) that associates a unique POVM with the empirical predictions for measurement statistics.

We begin with a brief review of Bohmian mechanics and quantum equilibrium before providing a detailed elaboration of the POVM theorem in the central Section 3. Section 4 provides additional discussion of the assumptions. Section 5 presents two key examples for POVMs—describing a position measurement and an indirect measurement, respectively—that illustrate applications of the general theorem. We conclude with Section 6, which discusses the scope of the POVM theorem and offers clarifications on Questions 3 and 4.

As a preliminary answer to Question 2, we note that the assumptions of the POVM are not intended to add general principles to the Bohmian laws but to specify what counts as a “measurement” in the sense of the theorem. It is precisely because “measurements” do not occupy a fundamental place in the theory that such a specification is necessary.

## 2. Bohmian Mechanics and Quantum Equilibrium

While an analogous result may also be obtainable in other quantum theories (for example, Tumulka [12] formulates a POVM theorem for GRWf, the GRW collapse theory [13] with flash ontology [14,15]), we will present the POVM theorem as a theorem in Bohmian mechanics, where it is conceptually clearest and where the aforementioned controversy has, nonetheless, arisen.

Bohmian mechanics (BM) is a theory about particles whose motion is guided by the wave function. The state of a Bohmian system is described by its wave function $\psi_{t}$ and the actual configuration Q(t) of its constituent particles. The fundamental dynamical laws are the Schrödinger equation for the wave function,

(2) $$i\partial_{t}\psi_{t}\left(q\right)=H\psi_{t}\left(q\right)$$

and the guiding equation for the particles,

(3) $$\frac{dQ(t)}{dt}=v^{\psi_{t}}\left(Q\left(t\right)\right)$$

with the vector field

(4) vψt(q,t)=ħmIm∇ψt(q)ψt(q).

For spinor-valued wave functions, (4) straightforwardly generalizes to vψ=ħmImψ†∇ψψ†ψ, though it is natural to add an additional spin-dependent term that results from the non-relativistic limit of the Dirac current [16].

On the fundamental level, BM involves only one wave function, the *universal* wave function $\Psi_{t}$ that guides all the particles together. The wave function of a subsystem (with configuration coordinates *x*) is the *conditional wave function*

(5) $$\psi_{t}^{Y(t)}\left(x\right)=\frac{\Psi_{t}\left(x,Y\left(t\right)\right)}{\sqrt{\int {\left|\Psi_{t}\left(x,Y\left(t\right)\right)\right|}^{2}\;dx}},$$

where Y(t) is the actual configuration of the environment, i.e., the rest of the universe.

Under certain conditions (that are, per se, special but routinely created in experimental situations), the dynamics of a subsystem decouple from the environment and can be described as an independent Bohmian system whose wave function follows an autonomous Schrödinger equation. These conditions are met if Hamiltonian interactions between the *x*- and *y*-system are negligible and the universal wave function is of the form

(6) $$\Psi_{t}\left(x,y\right)=c_{1}\varphi_{t}\left(x\right)\Phi_{t}\left(y\right)+c_{2}\Psi_{t}^{⊥}\left(x,y\right),$$

where $\Phi_{t}$ and $\Psi_{t}^{⊥}$ have (essentially) disjoint *y*-support and $Y\left(t\right)\in \mathrm{supp}\left(\varphi_{t}\right)$. In this case, the conditional wave function of the *x*-system becomes the *effective* wave function

(7) $$\psi_{t}^{Y_{t}}\left(x\right)=\frac{\Psi_{t}\left(x,Y\left(t\right)\right)}{\sqrt{\int {\left|\Psi_{t}\left(x,Y\left(t\right)\right)\right|}^{2}\;dx}}=\varphi_{t}\left(x\right).$$

The statistical analysis of the Bohmian theory is based on the following.

**Quantum Equilibrium Hypothesis (QEH):** If a subsystem of the universe has (conditional) wave function $\varphi_{t}$, then the probability of its particle configuration $X_{t}$ at time *t* being in the volume element dx of its configuration space is given by
  (8) $$P^{\varphi_{t}}\left(X_{t}\in dx\right)={\left|\varphi_{t}\left(x\right)\right|}^{2}\;dx.$$

A system including particles with spin may not have a conditional wave function but only a conditional density matrix [17]. While the quantum equilibrium hypothesis extends to conditional density matrices (after tracing out the spin degrees of freedom), this will not be relevant to the following version of the POVM theorem, which assumes that the laboratory system is separable from its environment (and thus has an *effective* wave function).

It is important to appreciate that the QEH is not somehow restricted to “microscopic” systems (indeed, it would have little empirical content if it were) and that it does not in any way involve or presuppose “measurements”. In BM, the Born rule in the form of (8) refers to *actual* particle positions.

We take the quantum equilibrium hypothesis to be grounded in the fundamental dynamical laws of BM by the (Neo)-Boltzmannian analysis of DGZ [18]. It is then not an additional law or postulate but a *typical* regularity entailed by the Bohmian laws [19]. While the POVM theorem does not hinge on this view, understanding (8) as an *equilibrium* distribution—and that our universe, conceived as a Bohmian universe, *is* in quantum equilibrium—goes a long way towards understanding the strength of the theorem and why it reflects certain limits on our empirical access to the *primitive ontology* [15,18,20], i.e., the microscopic particle trajectories. An equilibrium situation always limits the kinds of correlations that may be obtained between two subsystems; here, between a microscopic target system and some macroscopic “apparatus” supposed to record information about the former (cf. the theorem of *absolute uncertainty* in [18]).

## 3. The POVM Theorem and Its Assumptions

We will now provide a systematic presentation of the POVM theorem and its underlying assumptions based on the analysis of the measurement process in Bohmian mechanics.

### 3.1. Basic Setting

We consider a *laboratory system* as a subsystem of the universe. This lab system is divided into a *target system* (coordinates $x\in R^{m}$, Hilbert space $H_{T}$) and an *apparatus system* (coordinates $y\in R^{n}$, Hilbert space $H_{A}$). The apparatus system may include more than the actual measurement devices (In fact, what we call the “lab system” need not be restricted to an actual laboratory). If we want, we can further divide the lab system into target system, apparatus, and environment (inside the lab system), but this will not be necessary for our analysis. $H_{T}$ need not be the full Hilbert space of the *x*-system; it can be a subspace of wave functions for which the experiment makes sense.

The apparatus system must have some degrees of freedom (we will refer to them as a “pointer”) that can indicate macroscopically discernible measurement outcomes. This means that there exist disjoint regions $V_{k}\subset R^{n}$ of its configuration space such that $Y\in V_{k}$ indicates the outcome “*k*”, usually associated with some number $\alpha_{k}\in R$. These outcomes may include a “null result”, $Y\in V_{0}$, corresponding to the pointer not moving at all. We denote this set of possible outcomes by A.

In [2,12], the coarse-graining $F\left(V_{k}\right)=\alpha_{k}$ (which can be understood as a macro-variable on configuration space, cf. [21]) is called *calibration function*. For simplicity, we will omit calibration functions for the most part and simply denote outcomes by their index k∈N (i.e., we set $\alpha_{k}=k$).

It is mathematically convenient (and physically meaningful) to consider a complete partition of the apparatus system’s configuration space by accounting for the possibility of a *failed* measurement, $Y\in R^{n}\setminus {⋃}_{k}V_{k}$. This corresponds to the case that the apparatus does not indicate any meaningful outcome (but breaks apart, for example). Of course, for a decent experiment, this should have a negligibly small probability. But a priori, the set of possible outcomes is, strictly speaking, $A^{\prime }=A\cup \left\{\mathrm{fail}\right\}$.

We focus on *discrete measurements*, meaning that the outcome set A (resp. $A^{\prime }$) is finite or countably infinite. The analysis can be extended to measurements with a continuum of possible outcomes. But no measurement device has an infinitely high resolution, and even if it did, no experimentalist could actually discern a continuum of pointer positions. Therefore, we hold that it is always legitimate to assume that the set of relevant outcomes is discretized.

Note that the notion of “outcome” here is purely instrumentalist; no assumption is made about what the outcomes mean, i.e., how they (more precisely, the outcome values given by the calibration function) correlate with physical quantities pertaining to the target system. Such considerations are, of course, relevant for apprehending the physical meaning of a measurement—and can be useful in determining a concrete expression for the associated POVM—but they do not affect the general theorem we are about to present. In fact, most quantum experiments do not measure any pre-existing property of the target system; the outcomes must rather be understood as the *product* of the measurement process involving both system and apparatus [2,14,21].

### 3.2. The Basic Theorem

We begin with the basic assumptions underlying the POVM theorem. Further remarks and justifications will be provided in Section 4.

**Assumption** **1** (External Influences are Negligible)**.** *Influences from outside the lab system can be neglected. This means that the lab system can be treated as a closed Bohmian subsystem with an effective wave function Ψ=Ψ(t,x,y) that undergoes a unitary Schrödinger evolution. The corresponding Hamiltonian H on $H_{T}⊗H_{A}$ involves, in particular, the interactions between the target system and apparatus system.*

**Assumption** **2** (State Preparation)**.** *The target system is prepared such that, at the initial time t=0 (the beginning of the measurement), its conditional wave function is an effective wave function $\varphi \in H_{T}$. Together with Assumption 1, this means that the total wave function of the lab system (including spin degrees of freedom) factorizes:*

(9) $$\Psi_{0}\left(x,y\right)=\varphi \left(x\right)⊗\Phi_{0}\left(y\right).$$

This initial state will evolve with the relevant unitary evolution $U_{t}=U\left(t,0\right)$ on $H_{T}⊗H_{A}$. It follows from (9) and the linearity of *U* that

(10) $$\Psi_{t}=U_{t}\Psi_{0}=U_{t}\left(\varphi ⊗\Phi_{0}\right)$$

depends linearly on φ, the initial wave function of the target system.

Assumptions 1 and 2 are sufficient to prove the following general version of the POVM theorem:

**Theorem** **1** (POVM theorem, general version)**.** *Let $F:R^{m}\times R^{n}\to R$ be a measurable function from the configuration space of the lab system into real numbers. For any fixed t≥0 and any (measurable) subset A⊆R, there exists a positive operator $E_{t}\left(A\right)$ (bounded by 1) acting on $H_{T}$ such that*

(11) $$P^{\Psi }\left(\left(X_{t},Y_{t}\right)\in F^{-1}\left(A\right)\right)=\int_{F^{-1}\left(A\right)}\;{\left|U_{t}\left(\varphi ⊗\Phi_{0}\right)\left(x,y\right)\right|}^{2}\;d^{m}x\;d^{n}y=\langle \varphi |E_{t}\left(A\right)\varphi \rangle .$$

*The map $E_{t}:A\mapsto E_{t}\left(A\right)$ defines a* Positive Operator-Valued Measure (POVM)*, i.e., it is normalized,*

(12a) $$E_{t}\left(R\right)={\mathrm{Id}}_{H_{T}},$$

*and countably additive*

(12b) $$E_{t}\left({⋃}_{j}A_{j}\right)=\sum_{j}E_{t}\left(A_{j}\right),\;\mathrm{for}\;\mathrm{pairwise}\;\mathrm{disjoint}\;A_{j}\subset R$$

*where a countably infinite operator sum is understood in the strong operator topology.*

In our setting, one should think of *F* as a coarse-graining function with $F(R^{m}\times V_{k})=k$; that is, pointer configurations $Y_{t}\in V_{k}$ are mapped to the outcome *k*. (While $F(Y\;\in \;R^{n}\setminus {⋃}_{k}V_{k})=\mathrm{fail}$, i.e., we have a POVM on the σ-algebra of $A^{\prime }$).

**Proof.**  Given $\Phi_{0}$ and a fixed t≥0, we define for any (measurable) A⊆R a sesquilinear form on $H_{T}⊗H_{T}$ by

(13) $$\begin{array}{cc} B_{t}\left[A\right] & \left(\varphi_{1},\varphi_{2}\right):=\int {\left(U_{t}\left(\varphi_{1}⊗\Phi_{0}\right)\right)}^{*}\;1_{F^{-1}\left(A\right)}\left(x,y\right)\;\left(U_{t}\left(\varphi_{2}⊗\Phi_{0}\right)\right)\;d^{m}x\;d^{n}y, \end{array}$$

and observe that $B_{t}\left[A\right]\left(\varphi ,\varphi \right)=\int_{F^{-1}\left(A\right)}{\left|U_{t}\left(\varphi ⊗\Phi_{0}\right)\left(x,y\right)\right|}^{2}d^{m}x\;d^{n}y=P^{\Psi }\left(\left(X_{t},Y_{t}\right)\in F^{-1}\left(A\right)\right)$. According to the *Riesz Representation Theorem*, there exists a positive operator $E_{t}\left(A\right)$ on $H_{T}$ such that $B_{t}\left[A\right]\left(\varphi_{1},\varphi_{2}\right)=\langle \varphi_{1}|E_{t}\left(A\right)\;\varphi_{2}\rangle$, which entails (11). The POVM properties (12) follow easily from $F^{-1}\left(R\right)=R^{m}\times R^{n}$ and $F^{-1}\left({⋃}_{j}A_{j}\right)={⋃}_{j}F^{-1}\left(A_{j}\right)$, for pairwise disjoint $A_{j}\subset R$. □

### 3.3. Towards Practical Predictions

Theorem 1 is a precise and very general mathematical result. It establishes the existence of POVMs encoding theoretical outcome distributions, which would typically approximate the corresponding empirical distribution for a series of repeated, independent trials. However, the POVMs derived in Theorem 1 may, in principle, depend both on the time *t* at which the pointer distribution is evaluated and on the initial wave function $\Phi_{0}$ of the apparatus system. In practice, the exact wave function of the apparatus system is beyond experimental control, and the time at which a measurement concludes may itself be random—particularly in cases involving a “passive” particle detection. A more basic point is that sensible predictions should not depend on the exact time at which the outcomes are read off. To associate concrete statistical predictions with a unique POVM, we therefore introduce two further assumptions about what constitutes a meaningful measurement experiment.

**Assumption** **3** (Stable Pointer Configurations)**.** *There exists some time interval I (on an empirically relevant time scale) during which the apparatus reliably indicates one—and only one—of the possible outcomes, i.e.,*

(14) $$\exists k\in A:Y_{t}\in V_{k},\forall t\in I$$

(with FAPP certainty). With Theorem 1, it follows that the probability for the recorded outcome *j* is given by the POVM element $E_{j}=E_{t}\left(j\right)$ for any t∈I. That is, from (11) with $F^{-1}\left(j\right)=R^{m}\times V_{j}$,

(15) $$P^{\Psi_{t}}\left(Y_{t}\in V_{j}\right)=\int_{y\in V_{j}}{\left|U_{t}\left(\varphi ⊗\Phi_{0}\right)\right|}^{2}\;d^{m}x\;d^{n}y=\langle \varphi |E_{j}\;\varphi \rangle =:P^{\varphi }\left(j\right).$$

The exact time at which the apparatus *begins* to display the measurement outcome may be random, but this is unproblematic as long as we can assume some (non-random) time frame *I* during which the apparatus still displays/records that same result.

The assumption of stable outcome configurations also provides further insight into the nature of the measurement process. A necessary condition for (14) (under Assumption 1 of a closed lab system) is that, for t∈I, the total wave function has the form

(16a) $$U_{t}\Psi_{0}\left(x,y\right)=\Psi_{t}\left(x,y\right)=\sum_{k}c_{k}\psi_{k}\left(t,x,y\right),$$

with constant coefficients $c_{k}\in C$, where the *pointer states* $\psi_{k}$ (each a normalized solution of the relevant Schrödinger equation) have (FAPP) disjoint *y*-support in $V_{k}$, i.e.,

(16b) $$\int 1_{V_{j}}\left(y\right){\left|\psi_{k}\left(t,x,y\right)\right|}^{2}\;d^{m}x\;d^{n}y\approx \delta_{jk},\;\forall t\in I.$$

This entails, in particular, that the $\psi_{k}$ are mutually (nearly) orthogonal with $\langle \psi_{k},\Psi_{t}\rangle \approx c_{k}$.

If condition (16b) did not hold, there would be a non-negligible probability that the apparatus does not indicate any meaningful outcome, or that the indicated outcome changes during the time interval *I*. Hence, a measurement process that reliably results in stable pointer configurations as outcomes must produce a stable branching of the wave function $\Psi_{t}$ into pointer states that remain localized in well-separated regions of configuration space.

With (16), we obtain a more transparent expression for (15):

(17) $$\begin{array}{cc} P^{\varphi }\left(j\right) & =\langle \varphi |E_{j}\;\varphi \rangle =P^{\Psi_{t}}\left(Y_{t}\in V_{j}\right)=\int_{y\in V_{j}}{\left|U_{t}\left(\varphi ⊗\Phi_{0}\right)\right|}^{2}\;d^{m}x\;d^{n}y\\ \; & =\int_{y\in V_{j}}{\left|\sum_{k}c_{k}\psi_{k}\left(t,x,y\right)\right|}^{2}\;d^{m}x\;d^{n}y\approx \int_{y\in V_{j}}{\left|c_{j}\psi_{j}\left(t,x,y\right)\right|}^{2}\;d^{m}x\;d^{n}y={\left|c_{j}\right|}^{2}. \end{array}$$

The approximation follows from (16b) and the Cauchy–Schwarz inequality

$${\left|\int_{y\in V_{j}}\psi_{k}^{*}\psi_{l}\;dz\;\right|}^{2}\leq \int_{y\in V_{j}}|\psi_{k}{|}^{2}\;dz\int_{y\in V_{j}}{\left|\psi_{l}\right|}^{2}\;dz\approx \delta_{jk}\delta_{jl}.$$

The upshot is that the outcome probabilities are essentially determined by the (coefficients of the) branching of the total wave function into the well-localized pointer states $\psi_{k}$. This branching (more than the resulting operators) is physically characteristic for a given measurement process.

**Remark** **1.** *We can provide a more explicit expression for the POVM elements (15) by writing*

(18) $$\begin{array}{cc} \langle \varphi |E_{j}\;\varphi \rangle & =\int_{y\in V_{j}}{\left|U_{t}\left(\varphi ⊗\Phi_{0}\right)\right|}^{2}\;d^{m}x\;d^{n}\;y\\ & =\int \left({\left(\varphi ⊗\Phi_{0}\right)}^{*}U_{t}^{†}P_{V_{j}}U_{t}\left(\varphi ⊗\Phi_{0}\right)\right)\left(x,y\right)\;d^{m}x\;d^{n}\;y\\ & =\int d^{m}x\;\varphi^{*}\left(x\right)\int d^{n}y\;\Phi_{0}^{*}\left(y\right)U_{t}^{†}P_{V_{j}}U_{t}\left(\varphi ⊗\Phi_{0}\right)\left(x,y\right), \end{array}$$

*where $P_{V_{j}}$ is the position projection operator on $H_{T}⊗H_{A}$ into the configuration space region $R^{m}\times V_{j}$ (corresponding to the indicator function $1_{V_{j}}$ in position representation). We thus read off*

(19) $$\left(E_{j}\varphi \right)\left(x\right)=\int \Phi_{0}^{*}\left(y\right)\;U_{t}^{†}P_{V_{j}}U_{t}\Phi_{0}\left(y\right)\;\varphi \left(x\right)\;d^{n}y.$$

*While this formula provides an explicit definition of the POVM elements rather than an abstract existence result, it is, by itself, of limited practical use since we generally have very limited access to the apparatus-system wave function $\Phi_{0}$ or even the full details of the time evolution $U_{t}$. However, more workable expressions (without explicit dependence on $\Phi_{0}$) can be derived for several more concrete measurement schemes (see, e.g., [4,22]). Two instructive examples are discussed in Section 5.*

**Remark** **2.** *In Bohmian mechanics, the outcome of the measurement is determined by the actual configuration $Y_{t}$ of the apparatus system, e.g., $Y_{t}\in V_{j}$. This also means that the Bohmian configuration determines one branch of the superposition (16a) as actually guiding the system. This is associated with a state transformation of the target system, usually involving a* collapse *of its conditional wave function (sometimes called* effective collapse *in the literature). While important, this aspect of the measurement analysis is beyond the scope of our discussion—which focuses on the outcome distribution—and we refer to [2,11,18,22] for details on how to describe the post-measurement state of the target system.*

The outcome probabilities encoded in the POVMs derived in Theorem 1—or (17) with the additional assumption of stable pointer configurations—depend (inter alia) on $\Phi_{0}$, the initial wave function of the apparatus system. That is, strictly speaking, we have $E_{j}=E_{j}\left[\Phi_{0}\right]$. A meaningful measurement is supposed to probe the target system and allow us to treat the degrees of freedom of the apparatus system as “control variables”. In practice, however, our control over the apparatus/laboratory wave function is very limited. As we repeat a measurement to obtain an empirical distribution of outcomes (i.e., statistics), the state of the lab system will never be *exactly* the same across different runs. Similar worries apply to the Hamiltonian that guides its time evolution. We can reset and re-gauge our instruments and control certain environmental factors (temperature, humidity, etc.) if they seem relevant, but at some point, we have to trust that nature is kind enough that the measurement results will not depend on the exact microstate of the laboratory or on what socks the experimentalist is wearing that day. If they did, experimental science would not be possible. To make these considerations explicit, we introduce the final assumption:

**Assumption** **4** (Apparatus Ready State as a Macrostate)**.** *As we repeat the measurement with the same or a different initial state φ of the target system, the initial state $\Phi_{0}$ of the apparatus system—and the interaction Hamiltonian that determines the unitary time evolution $U_{t}$—can be reproduced well enough that the form of the decoherent branching (16a) (and thereby the outcome probabilities) depends only on φ, and not on the residual variations in $\Phi_{0}$ (or $U_{t}$).*

*We formalize this assumption as follows: Let $V_{\mathrm{ready}}\subset R^{n}$ denote the set of apparatus system configurations $Y_{0}$ for which all instruments are properly reset. There exists $M_{0}\subset H_{A}$ (which need not be a linear subspace, although it arguably will be) such that*

*1.*  *All $\Phi_{0}\in M_{0}$ have FAPP support in $V_{\mathrm{ready}}$, i.e., $\int_{V_{\mathrm{ready}}}{\left|\Phi_{0}\left(y\right)\right|}^{2}\;d^{n}y\approx 1$;*
*2.*  *For all $\Phi_{0}\in M_{0}$, the decoherent branching (16a) has (essentially) the same form*
  (20) $$U_{t}\left(\varphi ⊗\Phi_{0}\right)\left(x,y\right)=\sum_{k}c_{k}\psi_{k}\left(t,x,y\right)$$
  *with coefficients $c_{k}$ depending only on φ and $\int_{R^{m}\times V_{k}}{\left|\psi_{k}\right|}^{2}\;d^{m}x\;d^{n}y\approx 1$ (The form of the pointer states $\psi_{k}$ may vary as long as they remain well-localized in $V_{k}$).*

*We call the macrostate $\left\{\left(Y_{0},\Phi_{0}\right):Y_{0}\in V_{\mathrm{ready}},\Phi_{0}\in M_{0}\right\}$ the* ready state *of the apparatus system and assume that the same ready state is prepared in each run of the experiment.*

This allows us to write

(21) $$E_{j}\left[\Phi_{0}\right]=E_{j}\left[M_{0}\right]$$

and infer that the POVM, as characterized in (15) and (17), will encode the typical outcome statistics of a series of repeated measurements.

**Remark** **3.** *Another subtle issue in moving from theoretical probabilities to statistical predictions is the* statistical independence *of repeated trials of a measurement (with the same or a different initial wave function φ of the target system). With the quantum equilibrium analysis of DGZ, statistical independence can, in fact, be proven for ensembles of subsystems prepared with an effective wave function [18] (Sections 5 and 10), i.e., follows from our Assumption 2. However, the details of the arguments are somewhat sophisticated and shall not be reproduced here.*

We can now state our final result associating the outcome statistics of an experiment with a POVM acting on the Hilbert space of the target system.

**Theorem** **2** (POVMs as statistical bookkeepers)**.** *Under assumptions 1–5, BM predicts that the empirical outcome distribution of a series of repeated measurements is—to a very good approximation—described by a POVM on the σ-algebra of A with operators acting on $H_{T}$, the relevant Hilbert space of initial wave functions of the target system.*

## 4. More on the Assumptions

### 4.1. On Assumption 1: The “Lab” as a Closed System

1. In BM, “external influences” may arise either from interactions on the level of the wave function or from the nonlocal guiding equation when the lab system is entangled with its environment (the former typically implies the latter, but not vice versa). Assumption 1 amounts to requiring that both are negligible for predicting the outcome statistics of the experiment.
2. Of course, everything that has a non-negligible influence on the outcome statistics should be included in the “laboratory system”, which can be defined as large as necessary to make the assumption plausible.
3. If the experiment involves measurements on entangled particles performed in different laboratories, then the “lab system” must comprise all of these individual laboratories even if they are located far apart.
4. Das and Aristarhov [10] object that, in practice, no laboratory subsystem is perfectly closed. Indeed, as with essentially all theoretical predictions, the POVM theorem is derived under idealized measurement conditions. The relevant question is then: does the fact that no laboratory is ever perfectly isolated only introduce noise, or can it change the “signal” into some non-POVM distribution? We see no reason to believe the latter (see point 8 below), while accounting for noise or potential measurement errors due to false detections is standard in experimental physics and beside the point of the theorem.
5. One might be, nonetheless, unsatisfied with the fact that Assumption 1 is an idealization rather than a strict truth claim about typical experiments. There are then two ways to relax the assumption, leading to variations of the theorem presented here.
6. One can take the “lab system” to be the entire universe [9], and thus Ψ the universal wave function, which always obeys a linear Schrödinger equation according to the fundamental laws of BM. However, in this case, the quantum equilibrium measure ${\left|\Psi \right|}^{2}$ should not be interpreted as a *probability* distribution but rather as a *typicality measure*, and the POVM theorem as a typicality result [19]. In short: for the vast majority of possible initial configurations of the universe (consistent with the experiment in question), the outcome statistics of a long series of measurements will be close to a POVM distribution. If the status of the QEH is understood following DGZ [18], probability statements are, in any case, grounded in these kinds of typicality statements, and the “universal” version of the POVM theorem is both natural and straightforward.
7. One can describe the lab system as an open quantum system in terms of a density matrix following a linear (e.g., Lindblad-type) master equation. The resulting version of the POVM theorem would be similar to the Choi–Kraus theorem in standard quantum mechanics [23,24]. This is in line with results implying that the Bohmian conditional wave function—undergoing effective collapses due to interactions with a large environment—typically behaves like the wave function in some spontaneous collapse model [25,26].
8. While a detailed discussion is beyond the scope of this paper, it is instructive to explain briefly why the results of our analysis hold up in the setting of open quantum systems. The entanglement of the lab system with its environment has essentially two effects. First, it will introduce some noise into the evolution of Ψ, which would now have to be described as a conditional (not effective) wave function. However, this noise is effectively random and will not skew the outcome statistics. Second, the lab wave function, after the primary measurement interaction, would not persist in a superposition (16a) but quickly collapse into the “pointer state” belonging to the actual outcome. Crucially, though, the corresponding “collapse probabilities” coincide (essentially) with the outcome probabilities that we are going to derive for a closed system. Averaging over the environment configurations (with respect to the quantum equilibrium measure) leads to the aforementioned description in terms of a (conditional) density matrix.

### 4.2. On Assumption 2: State Preparation

1. From Assumptions 1 and 2, it follows that at initial time $t_{0}$, the apparatus system can also be described by its own (effective) wave function $\Phi_{0}\in H_{A}$. Of course, as soon as the target system and apparatus system begin to interact, they will become entangled and no longer have separable quantum states.
2. While it is hard to justify from first principles why subsystems can, in fact, be prepared in separable quantum states, the Bohmian concept of an effective wave function (see [18] and our brief summary in Section 2) provides a handle on the issue that is not available in other quantum theories.
3. Uncertainty due to unreliable state preparation can be handled in the usual way by describing the state of the target system by a density matrix representing a (proper) mixture of pure states.

### 4.3. On Assumption 3: Stable Pointer Configurations

1. Our Assumption 3 required stable pointer configurations (during some time interval *I*) that record the final outcome of the measurement. From this assumption, we inferred the decoherent branching (16) of the lab wave function according to well-localized pointer states. At the same time, there are strong independent reasons to expect that the pointer states will decohere extremely well and fast due to the macroscopically large number (∼$10^{25}$) of degrees of freedom of the “pointer” and its environment inside the lab [27]. Put simply, once the $\psi_{k}$ separate on the extremely high-dimensional configuration space of the lab system, the dynamics will not bring them back into overlap (on empirically relevant timescales). In general, this separation of the pointer states gets only better (i.e., decoherence is stronger) the larger the system that we consider as our lab system.
2. In Bohmian mechanics (with the standard guiding equation), it is highly plausible that (16) entails (the typicality of) stable pointer configurations because trajectories typically avoid regions of configuration space where ${\left|\psi \right|}^{2}$ is vanishingly small. Realistically speaking, different pointer states $\psi_{k}$ will have highly suppressed but non-vanishing tails (which overlap outside the disjoint configuration space regions $V_{k}$). It is then not *impossible* for the apparatus configuration to quickly evolve from $Y\in V_{k}$ to $Y\in V_{j\neq k}$. However, those would be atypical solutions of the kind that all known microphysical theories admit in principle—solutions corresponding to, say, a pointer pointing to “1”, spontaneously evaporating, and reassembling to point to “2”.
3. That said, we emphasize that our analysis in Section 3.3 assumes stable pointer configurations rather than inferring them. If the measurement does not result in any reasonable stable, macroscopically discernible outcome, we simply do not have a meaningful experiment.
4. A bit of pragmatism is required to identify a suitable time interval *I* during which we can assume a stable record of the measurement outcome for a given experiment. As mentioned earlier, *I* need not include the exact time (from the start of the measurement) at which a detector is triggered—since this might vary across different runs of the measurement—as long as we can identify a (non-random) time interval during which some part of the apparatus configuration (e.g., the clock in an arrival time experiment) still records the relevant outcome. On the other hand, if the experiment involves, say, radioactive decay of ^235^U, decay events may occur even after a billion years. However, since it is difficult to obtain funding for an experiment that runs for a billion years, one will, in practice, cut off the tail end of the “waiting time distribution” much sooner—that is, agree on a much earlier time at which the experiment is considered concluded.
5. In principle, the relevant “pointer configurations” need not even be a part of the measurement apparatus in the narrow sense. One could consider any (more permanent) record of the outcomes (e.g., a printout on a sheet of paper) as long as they are recorded in some positional configuration (cf. [28] (Ch. 4)).

### 4.4. On Assumption 4: Apparatus Ready State as a Macrostate

1. While this assumption would be very difficult to prove from fundamental principles (due to the complexity of any macroscopic lab system), it can be empirically corroborated. Repeat the experiment—not just a single measurement, but the whole series of measurements—at different times and/or places. If the outcome distributions are (approximately) the same, it is good evidence that they do not depend significantly on details of $\Phi_{0}$ that are beyond the experimenter’s control.
2. For *projective measurements*, the target system will have corresponding eigenstates $\varphi_{k}\in H_{T}$ for which one can require $P^{\varphi_{k}}\left(j\right)=\langle \varphi_{k}\;,\;E_{j}\left[M_{0}\right]\;\varphi_{k}\rangle =\delta_{jk}$, i.e., that the apparatus system can be prepared in a ready state such that it will (almost certainly) indicate the outcome “*k*” whenever the target system is prepared in the initial state $\varphi_{k}$. This requirement is *stronger* than our Assumption 4 (though it can be more directly empirically corroborated).

## 5. Examples

In this section, we discuss two instructive examples for more concrete measurement schemes that illustrate applications of the general POVM theorem. The first describes a position measurement and indicates why the position of a Bohmian particle at a given time can be accurately measured. Then, we derive the POVM of an indirect measurement, which provides the prototypical example of a non-projective measurement that cannot be described by a PVM, i.e., a textbook “observable”.

### 5.1. Measurement of a Particle Position

We want to derive the POVM for an (idealized) position measurement on a particle with (effective) wave function $\varphi_{t}\left(x\right)\in L^{2}\left(R^{3}\right)$ that evolves according to the free Hamiltonian $H_{0}$. Technically, the quantum equilibrium distribution (8) already corresponds to a (continuous) POVM, but the goal of this exercise is to present a scheme for a measurement performed by a macroscopic apparatus that yields a coarse-grained reading of the particle’s location.

We thus partition $R^{3}$ into countably many disjoint regions $R_{k},k\in N$. According to the QEH, the probability of the particle being in the region $R_{k}$ at time *t* is

(22) $$P^{\varphi }\left(X_{t}\in R_{k}\right)=\int 1_{R_{k}}\left(x\right){\left|\varphi_{t}\left(x\right)\right|}^{2}\;d^{3}x=\langle \varphi_{t}|P_{R_{k}}\varphi_{t}\rangle .$$

We assume that our position measurement occurs at time t=τ and is practically instantaneous. The measurement interaction entangles the particle and apparatus and results in a branching of their joint wave function of the form

(23) $$\varphi_{0}\left(x\right)⊗\Phi_{0}\left(y\right)\to \Psi \left(\tau ,x,y\right)=\sum_{k}\left(P_{R_{k}}\varphi_{\tau }\left(x\right)\right)⊗\Phi_{k}\left(y\right)$$

where $\Phi_{k}$ is well-localized in a region $V_{k}$ of the detector’s configuration space, so that $Y\in V_{k}$ indicates that the particle was detected in the region $R_{k}$. This branching of the lab-system wave function is analogous to (16a) with

(24) $$\begin{array}{cc} \; & \left(P_{R_{k}}\varphi_{\tau }\left(x\right)\right)⊗\Phi_{k}\left(y\right)=:c_{k}\psi_{k}\left(\tau ,x,y\right),\\ & {\left|c_{k}\right|}^{2}=\langle \varphi_{\tau }|P_{R_{k}}\varphi_{\tau }\rangle =\langle \varphi_{0}|e^{i\hbar H_{0}\tau }P_{R_{k}}e^{-i\hbar H_{0}\tau }\varphi_{0}\rangle . \end{array}$$

For t>τ, the pointer states $\psi_{k}\left(t,x,y\right)$ will continue to evolve. They will not remain localized in the *x*-coordinates but maintain their *y*-support in $V_{k}$ for an extended time interval *I* since the detector must provide some stable outcome record. As argued before, the weights ${\left|c_{k}\right|}^{2}$ will also remain (essentially) constant.

From (24), we see that the POVM describing the outcome statistics is simply given by

(25) $$E_{k}=e^{i\hbar H_{0}\tau }P_{R_{k}}e^{-i\hbar H_{0}\tau },$$

where $P_{R_{k}}$ is the projection onto the region $R_{k}$ (so the POVM here is a PVM), and the outcome probabilities $P^{\varphi }\left(k\right)=\langle \varphi |E_{k}\;\varphi \rangle$ coincide with the Born probabilities (22) (for the particle position at t=τ).

However, how do we know that the detector reliably indicates the actual particle position in any individual measurement? This follows from the separation of the pointer states (23) as they become entangled with the particle. Since $\psi_{k}\left(\tau ,x,y\right)$ has (FAPP) *x*-support in $R_{k}$ and *y*-support in $V_{k}$, we find that the probability of a false detection ($X_{\tau }\in R_{i}$ but $Y_{\tau }\in R_{j}$ for j≠i) is

(26) $$P^{\Psi_{\tau }}\left(X_{\tau }\in R_{i},Y_{\tau }\in V_{j}\right)=\int_{R_{i}\times V_{j}}{\left|\sum_{k}c_{k}\psi_{k}\left(\tau ,x,y\right)\right|}^{2}\;dx\;dy\approx 0.$$

Note that while we can compute the outcome probabilities from the stable pointer configurations at any time t∈I, we did apply the QEH at t=τ to see the correlation between particle position and pointer configuration.

### 5.2. Indirect Measurements

Prototypical examples of non-projective measurements are provided by the indirect measurement scheme. An indirect measurement is a composite (two-part) measurement in which the target system first interacts—and thus becomes entangled—with a microscopic part of the apparatus system (probe/ancilla). The probe is then measured projectively, resulting in a macroscopic record. This scheme encompasses a wide range of physically relevant measurements, including weak measurements (without post-selection, see Section 6.1), which are basically indirect measurements with very extended and overlapping probe states. Arguably, every measurement is, at the end of the day, an indirect measurement since the target system first interacts with some microscopic part of the apparatus (e.g., excites or ionizes atoms), before an amplification mechanism provides a macroscopic “readout” of this primary interaction. Moreover, according to *Naimark’s dilation theorem* [29,30,31], every non-projective POVM can be derived from a projective measurement on a larger Hilbert space, corresponding to the readout of an indirect measurement scheme (although this abstract mathematical construction may not always be physically meaningful).

In an indirect measurement, the measurement interaction *U* (for ease of notation, we omit the time indices) consists of the following two parts: a pre-measurement (PM) interaction $U_{\mathrm{PM}}$ between the target system and the probe system, and the readout $U_{\mathrm{RO}}$ of the probe by the macroscopic apparatus (which, for simplicity, we assume to be a non-degenerate ideal measurement).

We consider an initial quantum state of the target system $\varphi \in H_{T}$ and an ONB $\left\{\varphi_{k}\right\}\subset H_{T}$ such that $\varphi =\sum_{k}a_{k}\;\varphi_{k}$ with ak=φkφφφk. We assume that there is a “ready state” $\chi_{0}^{P}\in H_{P}$ of the probe system (with generic configuration coordinates $y_{P}$) and a set of (not necessarily complete or orthogonal) “marker states” $\left\{\chi_{k}^{P}\right\}\subset H_{P}$ such that the PM interaction yields

(27) $$U_{\mathrm{PM}}\;\varphi_{k}\;\chi_{0}^{P}=\varphi_{k}\;\chi_{k}^{P}.$$

Since the *readout* is a projective (here even ideal) measurement of the probe system, there exists an ONB $\left\{\phi_{l}^{P}\right\}\subset H_{P}$ of eigenstates such that

(28) $$U_{\mathrm{RO}}\;\phi_{l}^{P}\;\Phi_{0}^{A}=\phi_{l}^{P}\;\Phi_{l}^{A},$$

where $\Phi_{0}^{A}$ is the initial state of the apparatus, and $\Phi_{l}^{A}$ pointer states corresponding to the final outcomes *l*. We can express the probe-marker states in terms of the probe-ONB as $\chi_{k}^{P}=\sum_{l}b_{kl}\;\phi_{l}^{P}$ with bkl=ϕlPχkPχkPϕlP. We assume that the three systems (target system, probe, and apparatus/lab system) are independently prepared so that we can start again with a product wave function.

The resulting measurement process thus has the following form:

(29) $$\begin{array}{cc} \varphi \left(x\right)\;\chi_{0}^{P}\left(y_{P}\right)\;\Phi_{0}^{A}\left(y_{A}\right)= & \left(\sum_{k}a_{k}\;\varphi_{k}\left(x\right)\right)\;\chi_{0}^{P}\left(y_{P}\right)\;\Phi_{0}^{A}\left(y_{A}\right)\\ \overset{U_{\mathrm{PM}}}{⟶} & \left(\sum_{k}a_{k}\;\varphi_{k}\left(x\right)\;\chi_{k}^{P}\left(y_{P}\right)\right)\;\Phi_{0}^{A}\left(y_{A}\right)\\ \overset{U_{\mathrm{RO}}}{⟶} & \sum_{k}a_{k}\;\varphi_{k}\left(x\right)\left(\sum_{l}b_{kl}\;\phi_{l}^{P}\left(y_{P}\right)\;\Phi_{l}^{A}\left(y_{A}\right)\right)\\ = & \sum_{l}\left(\sum_{k}a_{k}\;b_{kl}\;\varphi_{k}\left(x\right)\right)\;\phi_{l}^{P}\left(y_{P}\right)\;\Phi_{l}^{A}\left(y_{A}\right). \end{array}$$

It is instructive to compare this with (16a) by incorporating the probe as a part of the apparatus system. With $y=(y_{P},y_{A})$, we see that the decoherent branches on the right-hand side of (16a) are now given by

$$c_{l}\;\psi_{l}\left(t,x,y\right)=\left(\sum_{k}a_{k}\;b_{kl}\;\varphi_{k}\left(x\right)\right)\;\phi_{l}^{P}\left(y_{P}\right)\;\Phi_{l}^{A}\left(y_{A}\right),$$

where the $\Phi_{l}^{A}$ are localized in disjoint regions $V_{l}$ of configuration space. Here, *t* is a time shortly after the readout is concluded and supposed to lie in the interval *I*, during which the final apparatus configuration is stable.

Setting $\Psi_{t}=\sum_{l}\left(\sum_{k}a_{k}\;b_{kl}\;\varphi_{k}\right)\;\phi_{l}^{P}\;\Phi_{l}^{A}$, the probability of $Y_{A}\in V_{j}$, i.e., the apparatus configuration indicating outcome *j* can be computed as

(30) PΨt(YA∈Vj)=∫dx∫dyP∫VjdyA∑l∑kakbklφk(x)ϕlP(yP)ΦlA(yA)2≈∫dx∫dyP∫dyA∑kakbkjφk(x)ϕjP(yP)ΦjA(yA)2=∑kakbkj2=∑kφkφφφkϕjPχkPχkPϕjP2=∑kφφkφkφχkPϕjPϕjPχkPϕjPχkPχkPϕjPφkφφφk=〈φ|∑kχkPPjχkPPjχkPχkPφkφkφkφk|φ〉=:φEjφEjφφ

with the projections Pj=ϕjPϕjPϕjPϕjP acting on $H_{P}$. The POVM elements are thus given by

(31) Ej=∑kχkPPjχkPPjχkPχkPφkφkφkφk.

$E_{j}$ is a projection if and only if χkPPjχkPPjχkPχkP=1 for one or several *k* while all the other coefficients are 0 (i.e., $\chi_{k^{\prime }}^{P}=\chi_{k^{\prime \prime }}^{P}=\cdots =\phi_{j}^{P}$ for some $k^{\prime },k^{\prime \prime },\cdots$ and χkPϕjPϕjPχkP=0 for all $k\neq k^{\prime },k^{\prime \prime }\cdots$). This would entail that at least one marker state of the probe is also an eigenstate of the readout. Otherwise, and more generally, $E_{j}$ is not a projection, meaning, in particular, that there is no corresponding eigenstate of the target system. In general, indirect measurements are thus described by a genuine POVM that does not correspond to a PVM, i.e., a textbook “observable”.

## 6. On the Scope of the POVM Theorem

Having discussed what the POVM theorem is, we want to clarify what the theorem is *not*. In particular, while the POVM theorem is certainly relevant to any discussion of “measurability” (of Bohmian quantities) or “empirical equivalence“ (between BM and other quantum theories), it is not per se a theorem about either of these concepts, which are somewhat elusive to begin with.

### 6.1. Measurability and Non-POVM Statistics

Theorem 2 applies to the pointer statistics of a (repeated) measurement and imposes stringent constraints on how they depend on the wave function of the target system (namely, like a sesquilinear form). However, there exist special types of quantum experiments whose (reported) outcomes are not constrained in this way. Non-POVM statistics can be easily produced by either post-selecting a sub-ensemble from a series of repeated measurements or by not repeating the same measurement at all— instead, tuning the apparatus differently depending on the initial state of the target system. While each individual measurement is still associated with some POVM (as per Theorem 1), the resulting statistics are not encoded in any single POVM (i.e., do not fall under Theorem 2).

A trivial example (for the second strategy) is presented as follows: Perform an *x*-spin measurement if the target particle is prepared in an *x*-spin eigenstate, and a *y*-spin measurement if it is prepared in a *y*-spin eigenstate (etc.). Each of these individual measurements is associated with the usual spin observable ($\sigma_{x},\sigma_{y}$, etc.), but the empirical statistics of this experiment will not be encoded in any (single) POVM.

Thus, in principle, non-POVM statistics are obtained by simple manipulations of “POVM measurements”. They are straightforward to incorporate into the quantum measurement formalism and of little interest when conducted in a purely ad hoc fashion. And yet, such procedures can lead to meaningful and useful experimental schemes that may extend the range of what one might consider “measurable”. Here are the most important ones:

- **Weak Measurements with Post-Selection:** The POVM Theorem 2 applies to the “pointer statistics” of weak measurements. However, in this class of experiments (first suggested in [32]), one knows that the quantity one seeks to measure is—by design—very weakly correlated with the final pointer position of the measurement device. The distribution of the so-called *weak values* is then not obtained directly from the pointer distribution but, simply put, after some ”data processing” that involves averaging and post-selection (according to the results of strong measurements performed after each weak interaction). In this way, it is possible to—in a certain sense—measure quantities, e.g., Bohmian velocities [33,34,35], whose distribution does not correspond to a POVM.
- **Non-linear Measurements** do not repeat the same measurement process independently of the state of the target system. Instead, the apparatus and thereby, in general, the measurement interactions (which determine $U(t,t_{0})$) are tuned depending on φ—either based on the initial state preparation or depending on outcomes of intermittent measurements. Such a setup deliberately violates our Assumption 4. As a result, the total wave function (10) will not depend linearly on φ across *different* instances of the measurement and the empirical statistics will not be given by a (fixed) POVM. (See our trivial example for spin measurements above). Interesting applications of non-linear/adaptive measurement techniques include state-dependent *protective measurements* [36] or adaptive quantum state tomography [37,38]. Weak measurements with post-selection may also be understood as a special type of non-linear measurement [22,34].

We said above that Bohmian velocities are measurable “in a certain sense”. What exactly does this mean? Well, if the question is whether some apparatus could reliably indicate the velocity of an individual Bohmian particle in an arbitrary state, then the answer is no. The proof is that the Bohmian velocity (3) and (4) does not depend sesquilinearly on the wave function, so its distribution cannot correspond to a POVM. However, the standard velocity field (4) can be reliably reconstructed from a series of weak measurements (with post-selection) on identically prepared particles [33,34,35]. An analysis of this experiment within BM (with the standard guiding equation) justifies an interpretation as a genuine (albeit non-standard) measurement of Bohmian velocities [22,34]. Notably, other quantum theories (including Bohm-type theories with modified guiding equations) predict the same outcome statistics—from which the standard Bohmian velocities are reconstructed—although, in those theories, the experiment may not be a measurement of anything.

This illustrates that the question of what is “measurable”—that is, whether and how the pointer readings of an experiment might be correlated with properties of the target system—is highly theory-dependent. Indeed, the question of whether Bohmian velocities can be measured arises only because Bohmian particles actually have a velocity that one might seek to measure. By contrast, as mentioned before, most quantum measurements do not reveal any pre-existing property of the target system anyhow [2,14,21].

The moral is that one must be careful if one wishes to read the POVM theorem as a “no-go result” for which quantities are measurable in Bohmian mechanics (or quantum mechanics, more broadly). “Measurability” is a subtle concept whose complete analysis lies beyond the scope of the theorem. Concretely: If a quantity associated with Bohmian systems is not intrinsically POVM-distributed (i.e., does not depend sesquilinearly on the wave function), this means that no apparatus could reliably reveal that quantity for an individual target system prepared in an arbitrary (or unknown) quantum state. It does not mean, however, that the quantity could not be “measurable” in a more roundabout, yet nonetheless meaningful, sense.

### 6.2. On “Empirical Equivalence”

The POVM theorem is a theorem about the outcome statistics of measurements in Bohmian mechanics. It also serves to ground the standard quantum measurement formalism in BM. However, it is not a theorem about the “empirical equivalence” of BM and standard quantum mechanics (QM) or any other class of quantum theories.

Claims to the contrary seem to take for granted that any quantum theory in which the Schrödinger equation and some form of Born rule for “positions” hold (at least approximately) must, in principle, predict the same outcome probabilities (11) or (17) that we derived within the Bohmian framework. However, whether this derivation, and its underlying assumptions, are justified in other quantum theories—where it might not even be clear what “apparatus configuration” means—is a separate question, requiring independent analysis. At a more basic level, the Born probabilities figuring into these equations may have different meanings in different quantum theories (e.g., in a Many-Worlds Interpretation), so it is not always evident that they carry the same empirical content.

While the POVM theorem undoubtedly plays a key role in clarifying the relationship between BM and standard QM, any straightforward answer to the question of empirical equivalence is already obstructed by the lack of consensus on what “standard quantum mechanics” even is. From a Bohmian point of view, a natural stance is that “standard quantum mechanics” is merely the effective measurement formalism that emerges from Bohmian mechanics. On this view, the very question of empirical equivalence is somewhat off-target, since BM and QM are not truly rival theories.

## Data Availability

Data are contained within the article.
